# Friluftsliv literacy—a contribution to physical literacy for health throughout the life course

**DOI:** 10.3389/fpubh.2023.1307444

**Published:** 2024-01-10

**Authors:** Eivind Sæther, Idar Lyngstad

**Affiliations:** Faculty of Education and Arts, Nord University, Bodø, Nordland, Norway

**Keywords:** physical literacy, friluftsliv, health, nature experience, quality of life

## Abstract

This article illuminates and discusses the concept “friluftsliv literacy” in relation to physical literacy for health throughout the life course. A literal translation into English of “friluftsliv” would be “free-air life” – or “outdoor life.” We obtained stories and interview data from older adult people who could help us by providing insights into friluftsliv literacy through a number of life phases. The research questions were: What can describe friluftsliv literacy from the perspective of experienced friluftsliv practitioners and generate understandings of friluftsliv literacy from childhood to old age, and how can friluftsliv literacy contribute to the conceptualization of physical literacy for health throughout the life course? We designed the study according to a constructionist research tradition and followed six analytical phases of reflexive thematic analysis of the interview data. We developed four themes from the data material and argue that friluftsliv literacy includes an existential phenomenological and idealist dimension in the way it offers a view on the active subject in friluftsliv through the various life phases and promotes nature experiences through the whole life. Moreover, friluftsliv literacy promotes “pure” friluftsliv experiences based on internal motivation and desire, voluntariness, and freedom. We also argue that throughout the life course friluftsliv literacy promotes important social values, especially within family contexts, social values that enhance the quality of life. Friluftsliv literacy also includes a pragmatic dimension and contributes to the conceptualization of physical literacy for health in the way that it promotes people learning to like friluftsliv at a young age, an attitude that can then be nurtured and maintained throughout one’s entire life by practising friluftsliv and adapting to individual capacities and life phases.

## Introduction

This work builds on an earlier study on the concept of “friluftsliv literacy” in relation to physical literacy in physical education pedagogies (1). The earlier study examined how the concept friluftsliv literacy can expand the concept of physical literacy in a useful manner, connecting it to identity formation, self-esteem and self-confidence within physical education in school. We argued that friluftsliv literacy supports existential and phenomenological perspectives on physical activity in accordance with Whitehead’s definition of physical literacy and provides key concepts relating to a physically literate person (2). According to Whitehead, a physically literate person will have the motivation, competence, and knowledge to appreciate and assume responsibility for taking part in meaningful physical activities throughout the life. Furthermore, a physically literate person will have a well-established sense of self as embodied in the world, and together with this have an articulate interaction with the environment. In addition, we argued in the study that friluftsliv literacy involves understanding and practising a social fellowship and participating in social groups in school, which a Whiteheadian approach to physical literacy does not provide so clearly. The concept friluftsliv literacy also problematizes the understanding of physical literacy based on the physical literacy discourse, which should relate more to concerns about the environmentally sustainable future, especially in these times of climate change (1).

Another point of departure for the study is that research literature on physical literacy makes few attempts to elaborate on physical literacy in the context of outdoor activities and moving about and being in nature – what we in the Norwegian context usually refer to as “friluftsliv.” This term has been established in the field and is now also used as an English term [see for example (1, 3–6)]. A literal translation into English of friluftsliv would be “free-air life” or the more recognizable “outdoor life.” Except our previous study (1), some connections to nature in questionnaires (7), and measurement instruments for physical literacy (8), it seems to be few attempts to conceptualize physical literacy in the context of nature and friluftsliv. Physical literacy is a concept that is applied in a wide range of settings, with different aims, approaches, and audiences (9). Even an alternative concept, movement literacy, has been promoted (10). It could be a cause for concern that the concept has taken many forms, encompassing different definitions, aims, and content, which might limit the capacity to draw meaningful conclusions regarding effective implementation in for example physical education contexts. Another concern could be that quality research in pedagogy might be limited by a lack of a clear understanding of the conceptual foundations of physical literacy (9). On the other hand, the fact that the concept physical literacy has taken many forms could be a source of creative and innovative debates, in which our study aims to contribute with a nature dimension and friluftsliv perspective in the discussion of physical literacy for health throughout life course.

We relate our current study to four physical literacy approaches in the research literature that range between dichotomous characterizations of “idealist” and “pragmatic” (11). One of these is the idealist embodiment approach, claiming that physical literacy develops the embodied human capability [e.g., (12)]. This is also called a Whiteheadian approach (13, 14). At the other end of the scale are the pragmatic health-determinant and the disease-approaches, which both claim to improve health behavior [e.g., (15)]. Between the idealist and two pragmatic approaches, Young et al. (11) define the idealist-pragmatic approach that positions physical literacy as an antecedent to physical activity [e.g., (16)] that also encompasses an embodied understanding (17). Young et al. (11) argue that the philosophical nature of the idealist approach to physical literacy can be positioned in contrast to the other three approaches that tend to promote a more pragmatic framing of physical literacy as a means to other ends. This modest position of a nature and friluftsliv perspective in the research literature is also apparent in other reviews of physical literacy literature, where three waves in the literature are described (18). One wave is physical literacy as health-promoting physical activity, and the second is physical literacy as motor competence, which can include a long-term athletic development approach with its focus on developing physical literacy through and for participation in sports (19). The third wave refers to physical literacy as phenomenological embodiment.

While the terms “nature” and “friluftsliv” have modest positions in a discussion on physical literacy, physical activity and health are concepts that are more or less permanently embodied in all physical literacy approaches. Young et al. (18) maintain that it is important to be open to different views on physical literacy, but at the same time argue that physical activity, quality of life, and health must be core areas in all the physical literacy waves. Our earlier study of friluftsliv literacy (1) presents arguments for friluftsliv that are connected to quality of life and health and may be relevant to discuss in relation to all approaches to physical literacy, ranging from the idealist approach to the pragmatic approach.

In another new review study that analyses research on physical literacy for the older adult people (20) the researchers summarize the research literature in this field as follows: findings from current literature indicate that engagement in purposeful, social, and diverse activities, obtaining knowledge of age-related changes, and being able to self-adapt to mobility fluctuations, is the foundation to becoming a physically literate adult [(20), p. 8149]. Petrusevski et al. argue that it is necessary to consider physical literacy more broadly than Whitehead’s approach and definition do; mobility and movement skills change as people move through the different life phases, and particularly in step with changes due to aging. But as mentioned above, the physical literacy literature provides few discussions based on a friluftsliv and nature perspective, and this appears to apply from an educational perspective, as well as in relation to physical literacy throughout the life course.

With this as our point of departure, our ambition has been to continue our research on the term friluftsliv literacy as it relates to several phases in people’s lives, not just within the context of physical education in school. We started a research process where we chose to obtain stories and data from retired people who could help us by providing insights into friluftsliv literacy through a number of life phases. Using key concepts of friluftsliv literacy from our previous study, which will be explained later in this article, our aim in the current study has been to explore the concept friluftsliv literacy from an expanded angle, involving people from 68 to 86 years of age. In the study, two women and two men, aged 68, 86, 68 and 75, reflected in interviews on their (long) lives as friluftsliv practitioners. The data material was collected to generate their retrospective and current views on friluftsliv throughout their lives. We sought to describe friluftsliv literacy throughout the life course by recruiting these experienced friluftsliv practitioners to take part in qualitative research interviews, which we entitled “friluftsliv from childhood to old age.” Another aim was to discuss friluftsliv literacy as a useful concept in relation to physical literacy for health throughout the life course. The research questions behind the process were: What can describe friluftsliv literacy from the perspective of experienced friluftsliv practitioners (aged 68–86) and generate understandings of friluftsliv literacy from childhood to old age? How can friluftsliv literacy contribute to the conceptualization of physical literacy for health throughout the life course?

We will further outline the Norwegian context and our phenomenological approach of the study. However, we start with a short discussion of our earlier study of the concept friluftsliv literacy, which focused on friluftsliv in school.

## The previous study and the concept “friluftsliv literacy”

In the previous study (1), we collected data from students in upper secondary school in Norway to generate two main themes that articulated key aspects of friluftsliv literacy: (1) experience of nature, sense of fellowship, and understanding of democracy, and (2) identity formation, self-esteem, self-confidence, and views on future life. The informants in the study related that the friluftsliv trips they took part in at their school had an emotional impact on them, that nature afforded them experiences, and that this took place in a fellowship where the students showed much trust in each other. The students’ stories gave us insight into friluftsliv that we related to their sense of self in the way it promoted self-confidence. The result of the analysis was in line with the notion of physical literacy, which outlines that students will come to learn something about themselves as movement subjects [(2), p. 19]. However, the students in our study reflected that they learned something about themselves as *subjects* in nature, not only learned various movement skills in friluftsliv. We interpreted that they learned “that an encounter with nature was more than a fleeting incident for them. It could be an important identity-formation element for their future growth and maturing” [(1), p. 520–521].

The students also experienced social collaboration processes and learned about democratic principles in fellowship with others. They experienced that this required the ability to cooperate and reflect on what one’s own contributions and efforts meant for the development of each student and the group on a friluftsliv trip. The results in the study indicate that students can learn that training to give their opinions, discussing various points of view, dealing with disagreements, and working to find acceptable common solutions can furnish them with vital social skills [(1), p. 8]. This social learning aspect is in line with the ideas behind a proposed social domain in the concept of physical literacy (21), which provides social skills for interaction with others, including communication, cooperation and conflict resolution, and the concept social capability when performing physical activities together with others (22). Our study corresponds with findings of social and collaborative skills and an improved self-concept in research on learning in so called natural settings in school, as well (23), and with benefits of social belonging, involvement and being made responsible in friluftsliv activities in physical education (24). Our study promotes the idea that friluftsliv may contribute to human flourishing (25), although we are aware that friluftsliv, on the other hand, may create unpleasant experiences and limit the human flourishing for some students in school. According to Durden-Myers and Whitehead (25), human flourishing refers to a disposition whereby individuals are considered to be thriving or living optimally and within an optimal range of human functioning. From our perspective, we argued in our study that moving about and being in nature over time, experiences of nature, and social fellowship, can create meaningful experiences that can contribute to human flourishing for students in school, when friluftsliv is provided in the way it was experienced by the students in our study [(1), p. 8].

## The Norwegian context of the study

The term “friluftsliv” was first used by the Norwegian poet Henrik Ibsen in 1859 in his poem “Paa Vidderne,” *On the heights* (26). The concept has thus existed in the Norwegian language for well over a century. The concept friluftsliv encompasses complex relations and practices ranging from daily walking, sustainable awareness and an esthetic experience of nature to skill-oriented adventurous journeys into remote uncultivated environments [(3), p. 290]. The Norwegian Ministry of Climate and Environment defines friluftsliv as “staying outdoors and being physically active during leisure time to have a change of environment and to experience nature” [(27), p. 10]. This definition of friluftsliv relates to moving and being moved in nature over a specific period of time, changing one’s environment, and having experiences of nature. A key part of friluftsliv is the experience of nature, which the official Norwegian definition emphasizes. Furthermore, friluftsliv can be understood as a philosophy of life and therefore as a “lifestyle” [(5), p. 97] that makes people grow as human beings through engaging in asking, searching, probing and testing – all actions that may involve challenges and pain [(3), p. 293]. We argue that Norway has a friluftsliv tradition, which means that there are dispositions or tendencies with regard to how people in Norway tend to think about friluftsliv and behave as friluftsliv practitioners. Norwegian friluftsliv can also be characterized by closeness and friendliness to nature, which result in continuing popularity of friluftsliv activities like shorter walks, day trips on foot and bike, swimming and sunbathing, and cross country skiing in nearby natural environment in the winter season [(3), p. 290]. Norwegian law provides access to nature areas for all the population through the so-called *allemannsretten* – the right to roam or the common right of access, even if several nature areas have been privatized or have restrictions on their use. Friluftsliv literacy may be affected by sociological factors regulating the performance of friluftsliv, such as gender, age, domicile, education, and economy (28, 29). People’s thoughts and behavior are results of socialization to and in friluftsliv within families, in school, among peers, and in organizations that promote friluftsliv [according to Bronfenbrenner (30)]. A broad understanding among Norwegian researchers on friluftsliv has been that the transfer of friluftsliv skills and their accompanying meaning systems may be seen as a socialization process [(28), p. 14; (31), p. 32]. The family is important in this transfer. Studies show that the family is an important socialization agent, even if other agencies, such as school, associations, and various media, also socialize to friluftsliv (28, 31). As in other countries, some people in Norway can see nature as dangerous, so that socialization processes may be affected negatively in some social settings. In addition, we have issues of crime and safety for some people to consider going alone or in small groups into nature, something that also may affect the friluftsliv activity. The Norwegian Ministry’s definition of friluftsliv implies that the idea behind it comprises more than physical activity. It refers to being inspired by and experiencing nature without necessarily practising much physical activity. Physical activity alone is thus an insufficient explanation of friluftsliv, even though physical activity *per se* takes place in many types of friluftsliv (1).

Bearing all these aspects in mind, the concept of friluftsliv is closely related to outdoor recreational activities. Contemplative aspects of friluftsliv combined with health promotion and physical activity constitute friluftsliv in the Norwegian context (32). Intrinsic values of friluftsliv are important through such ideas as freedom, the experience of nature, and quality of life, while friluftsliv is also considered to benefit the general population’s and the individual’s health. Norwegian official reports point to the great potential friluftsliv has for improving physical, mental, and societal health [(27), p. 9]. Thus, the perception of Norwegian authorities on friluftsliv includes both an idealistic view and a pragmatic view on friluftsliv (11). From the pragmatic perspective, friluftsliv is positioned as a means for increasing physical activity in the same way as physical activity is positioned as health promoting for the general population, as mentioned earlier. A meta-analysis of data from more than 226,000 people from various countries around the globe, including Norway, indicates that physical health benefits may be achieved by taking walks of more than 7,000 steps per day (33). Even short walks and a relatively low number of steps per day may have positive impact on physical health. Mental health can also be affected by friluftsliv (34). Helga Løvoll, professor in the friluftsliv field, states that esthetic experiences in nature may lead to more satisfied individuals in their day-to-day lives and may reduce stress (35). This might mean, for example, that being in a place out in nature and experiencing a starry night sky, glittering reflections in water, perhaps best in combination with the personal physical activity that got one to this place, can have significance for individuals. Løvoll claims that mastering nature can be associated with good mental health.

## The phenomenological approach of the study

A phenomenological approach to friluftsliv literacy creates an understanding of an active bodily subject, lived time and space, and individual meaning-formation in friluftsliv (1, 2, 10, 36–39). A view on an active bodily subject in friluftsliv refers to the individual as intentional and meaningful and that the individual senses, perceives, and experiences in friluftsliv activities [(1), p. 7]. This view provides a gateway to understanding an individual’s lived friluftsliv experience and the importance of these experiences “because the subjective, lived experiences provide us with a method for feeling, seeing, knowing, and understanding lived experience and the meaning(s) of those experiences” [(36), p. 27]. Lived time reflects the individual’s inner awareness of time and how past, present and future in the individual’s consciousness are connected and is an ongoing flow where what is ahead right now is experienced against a background linked to what has been and what will come in the future [(37), p. 70]. Lived space refers to the individual approach to friluftsliv activities and how he or she “inhabits” the nature environment when doing friluftsliv activities [according to Moran (38)]. Nature will be approached in different ways by individuals. For example, the weather and temperature may feel (uncomfortably) cold for some, while for others it does not feel that way. The individual can have an active and offensive – or a more defensive approach – to nature and friluftsliv activities. Meaning is formed by being mindfully engaged in doing something of personal importance (39). Meaning-formation is what shifts the individual from one experience to the next, and from an understanding of phenomena experienced in friluftsliv to a new or different understanding [according to Standal (10)]. The perception of meaning is also embodied (2), that is to say that in friluftsliv an individual experiences and forms meaning through the body. This applies to individuals of all ages, including the older adult people.

## Materials and methods

We interviewed four older adult people who have been active in friluftsliv throughout their lives. We were looking for stories and motifs that could describe their experiences of friluftsliv, stories that could tell us something about what friluftsliv has been for them, how they have practised friluftsliv, and the value friluftsliv has had through their many life phases. We were also looking for stories about what continues to create friluftsliv for them, what they think about friluftsliv today, and how friluftsliv is appreciated in the present. We were furthermore interested in data that could tell us something about what friluftsliv could mean for socialization with significant others in their lives, in the family or their circle of friends.

These four informants were handpicked because we believed that we would obtain stories and data material that could be used to illuminate and discuss friluftsliv literacy in different phases of life, from childhood to life as a pensioner. We assumed that these pensioners could tell us useful friluftsliv stories, which we did not know before we started our research process. The four pensioners were both homogeneous and heterogeneous. They all liked friluftsliv activities both in the local nature and in the wilderness. They had all practised friluftsliv throughout their lives, and were still active. We assessed that it strengthened our study to recruit older adult people up to 90 years of age to look back and reflect on their lifelong “friluftsliv.” At the same time, they had different experiences and special friluftsliv interests: One in hunting, one in fishing, one in botany, and one in social activity and community with other people in nature. Our handpicking of the four informants turned out to be a good choice as we obtained rich stories on friluftsliv from all four, stories from earlier phases of life and from their current lives.

The pensioners, whom we have called Mia, Peter, John and Eva, have lived all their lives in Norway, where there are distinct seasonal differences with cold, dark, and white winters, but mild, green, and sunny summers. The stories about their friluftsliv in Norwegian nature tell us about forests, lakes, rivers, mountains, the sea, woods, and paths that are found everywhere. They are also stories about being active outdoors during shifting weather conditions and temperatures, which requires them to consider which friluftsliv activities are suitable, which clothing to wear and the right equipment to have with them.

As the study comprises just a few selected interviewees the data has a narrow scope. It is therefore necessary to have some reservations about the area of validity of the stories we obtained from these four informants. However, the research project was designed according to and is situated within a constructionist research tradition (40), which opens for studies of phenomena in the lifeworlds and social phenomena of even a limited number of people, where understanding circumstances in individual lifeworlds is important (41). Constructionist epistemology frames the study, and we examine layers of meaning in the thoughts, reflections, and stories about friluftsliv throughout their lives. Researchers who use constructionist epistemology to frame their studies reconstruct narratives, stories, and data that are supplied by other people who are seated in their lifeworlds, generate thematic areas (42), and interpret and expand understandings that may have universal value and relevance (43).

Another fundamental aspect of the study’s scientific positioning is that the researchers themselves are subjects in the study, which thus constructs scientific knowledge through interpretation. The researchers encounter other people and phenomena in their field of study with a pre-understanding, which, as mentioned above, may influence the research process and the conclusions from the study (44). Researchers may encounter people in the study field with a set of preconceived opinions and attitudes that may have impact on the study. It is therefore necessary to have a self-critical attitude, openness, and transparency about the data collection and analytical processes if the interpretations are to be credible (41). In this study it should be mentioned that we have our own insights into friluftsliv, as we have many years of friluftsliv experience, including teaching friluftsliv and research work in the field. The first author has worked with friluftsliv fulltime for more than 30 years in university teacher education. The second author has worked with friluftsliv during three decades in university teacher education, although not fulltime. The second author has worked with the concept physical literacy in physical education in school, especially in a Norwegian context.

In addition to our own insights, practical experience and earlier research work, we had prior knowledge about these interviewees and their friluftsliv, as mentioned. We argue, however, that our pre-understanding of the friluftsliv of these four informants and our insights and experience beforehand may contribute to a deeper understanding of friluftsliv literacy and not to prejudiced conclusions. The four pensioners have something we do not have to the same degree, i.e., life experience from long lived lives, which turned out to be important when we settled on the themes (42) of friluftsliv literacy throughout the life course.

### Interview guide and conducting the interviews

The interview guide focused on key aspects of friluftsliv literacy from our previous study (1) and an existential and phenomenological approach to physical literacy (2). It was based on the categories of motivation, self-confidence, knowledge, skills, values, experiences of nature, health and friluftsliv, friluftsliv from childhood and through the various life phases to becoming a pensioner, fellowship through friluftsliv, and threats against nature. The interviews were conducted in the homes of the interviewees and lasted from 45 to 90 min. The first author conducted the interviews. As mentioned above, these interviews provided rich data on friluftsliv in the informants’ lives. All the four interviewees showed a very positive attitude to contributing during the interviews with high verbal skills. Our assessment is that the four interviews yielded a rich data quality relating to the value friluftsliv has had for them over several life phases, with reflections on friluftsliv today, and how it is valued in the present.

On two occasions we returned to two of the pensioners and asked them to elaborate on what they had said. This was done to clarify and elaborate on the understanding and meaning of two statements about, respectively, the experience of nature and so-called “pure” experiences of friluftsliv.

### Analysis and interpretation

We followed six analytical phases in line with Braun and Clarke’s phases of reflexive thematic analysis (42). The first phase was familiarizing ourselves with the dataset, where we read and reread the interview transcripts and made notes on analytical ideas. The second phase was coding of the data, including adding analytically meaningful descriptions – code labels – to these descriptions. We worked with coding on a latent level, which means that we captured the conceptual and implicit meaning of the stories we obtained from the interviewees. The analysis was inductive in part, which means that the focus was on the four interviewees’ experiences and memories of friluftsliv *per se*, without any use of theory concepts or lenses. At the same time, the analysis was deductive, which means that it was theory-driven in the way that it drew on key concepts of friluftsliv literacy from the previous study (1) and the existential and phenomenological definition of physical literacy (2). This combination of inductive and deductive analysis, often referred to as abductive analysis (45), allows us to be flexible in using (phenomenological) key concepts of friluftsliv literacy from our previous research as analytical lenses while remaining sensitive to constructing new insights without any theory concepts or lenses.

The third phase was to generate initial themes through an active analytical process. We constructed the themes based on the data, the research questions, and our knowledge and insights. Here, we identified candidate themes which in our assessment captured the data and addressed the research question. In phase four, we developed the themes once more after a reviewing process. We reflected on the nature of each theme, its core focus and its scope. We also started considering the relationship between the themes and existing key aspects of friluftsliv literacy. At the end of phase four, we started to consider the wider context of our research, focusing on the relationship between friluftsliv literacy and the concept physical literacy for health throughout the life course.

During phase five, we fine-tuned our analysis and ensured that each theme was clearly demarcated and built around the core concepts in our study. In this phase of the analysis, we defined and named the themes in addition to refining them.

The last phase of reflexive thematic analysis is, according to Braun and Clarke, writing the study. However, we started the writing process during phase three. We used familiarization notes and reflexive journalling to feed the writing process in this phase. Through phases three to six we had several meetings in which we discussed relevant data-interpretation alternatives. During phase six we weaved together our analytical “story,” using data extracts and finishing the writing process where our aim was to give readers a coherent and persuasive story about the dataset that addresses the research questions.

## Results

We developed four themes from the data material that describe friluftsliv literacy from childhood to old age and generate understandings of friluftsliv literacy in relation to physical literacy for health throughout the life course.

### Experiences of nature, identity formation, and memories that last

Mia, Peter, John, and Eva tell us about many nature adventures, experiences, and memories that have lasted. Friluftsliv changes from when you were a child, through your youth and adult life up to when you are a pensioner, John says. Friluftsliv shapes your identity at an early age. Lived life also affects and changes your identity, John states. He reflects that experiences are relative and must be considered in comparison to previous experiences and the aims a person has in wanting to have such experiences. John tells us that one time on a horse-riding trip he woke up to pleasant humming sounds when he spent a night with his companions in a Sami turf hut (*gamme*) in the mountains. One of the people in the tour group had a disability and this was the first time he had not “slept in his own bed.” This had been a “special experience” with many “impressions” they both recalled later. John says that he believes that this relates to “the importance of lived life for the life you get” and that “friluftsliv is part of this lived life.”

These pensioners also tell us that they all have been in situations that have been exciting, where perhaps some situations in winter have bordered on being dangerous. Or they have felt fatigued after a long hike. John (75) tells us about a winter trip where they “spent the night under open skies in the mountains, […] had lit a log fire, […] looked up at the starry skies, (and) […] heard the grouse chatting away in the surrounding hills.” John’s story is a description of living in and experiencing nature, including a starry sky in the dark of night, the heat of the fire, and the dusky grouse “talking together” in the cold winter mountains. The story inspires us to see man under the big sky as “small,” while the surrounding nature is “vast” and filled with life.

Peter (68) tells us about an adventure in pristine nature on a group of islands in the far north. He says he had a sense of being in a “zero zone.” He and his tour companions were skiing, and the landscape they were moving in was protected by strict heritage laws. There were no other people there. On the other hand, polar bears could be in the vicinity. Peter said it was “exciting” to feel “alone” while being aware that polar bears might be nearby. “Zero zone” means to be in the center of a special experience, but at the same time in a potentially hazardous place.

Mia (86) tells us about good experiences in light and pleasant weather in nature, but also about good experiences when there is “a lot of weather” (a typical Norwegian expression). “It can be nice in bad weather too…,” she says. She mentions how she experiences the skies, light, lakes, and high mountain plains as “beautiful.” Picking flowers and looking up the names of the plants are also good experiences, she states.

Eva (68) tells us about a winter trip where the snow was so cold, fine and light that it reminded her about “fluffy down feathers you could almost breathe in.” She described the snow as “special and fine and magical” and that she felt “joy, genuine joy over the beauty” in the fluffy snow.

The stories told by the four reflect experiences and memories ranging between macro- and micro-perspectives, between seeing oneself as small in the “vast” nature (under the stars and the night sky) and seeing the great (beauty) in little things (snow crystals weighing next to nothing), and that their experiences had been gained by moving out into nature, walking or skiing there, and getting away from the daily humdrum. They gave themselves the space and time to sense and experience nature, the star-filled night sky, the mountain plains, animal life and birds, the light, lakes, flowers, the heat of the log fire, the fluffy snow crystals, and having experiences that provide strong memories many years later.

### Social values and transfer of friluftsliv to new generations

Mia, Peter, John, and Eva tell us that they have spent time outdoors since they were very young. “I skied several miles from when I was three years old,” Mia tells us. Much of this activity took place without the parents being close by. But both the children and the parents were keen on being outdoors, she says. They learned to enjoy friluftsliv in their childhood.

Peter tells us that his interest in learning to like friluftsliv started with his father, “the officer,” “in the woods and mountains, skiing, tenting, and in lean-tos, learning to fish…” Because Peter chooses to mention his father, underlining “officer,” we think that there is something military, rhythmic, and rough about these trips. But this roughness made him “like” these activities right from the start. Peter adds that he was curious to discover new things from friluftsliv. Through his teens and student years his interest in friluftsliv grew strong, both because it was interesting in itself, but also because nature was somewhere to be social with others. “During my days at university we went on trips to remote log cabins with flirting and partying,” Peter says. All four informants found life partners with the same interests in friluftsliv as they had themselves. They found someone to be with who liked friluftsliv like themselves.

With their own family and children friluftsliv was what the family would do together: “We would rather take off into nature than go downtown,” John says. The trips in nature were a way of seeing their children develop and of creating socialization processes: “It was a nice trip when I walked with my son to the peak of Galdhøpiggen” (Norway’s tallest mountain), John states. His son was then eight years old, and John says he enjoyed seeing the motivation and progress of his young son. He says that “then I felt we had voluntarily taken a step further.” Taking “a step further” meant seeing his child reach a clear target and registering change in something considered to be important.

The four informants add that taking long walks with their children was not the most important thing. However, friluftsliv in the local environment was important. Eva says that when they had children, their friluftsliv had a local footprint, i.e., something that took place in nature close by to where they lived. This means that their friluftsliv was adapted to the age, capacity, interests and needs of the children.

An extra dimension was added to friluftsliv by being outside in nature with children and grandchildren. As a grandmother, Eva claims that “the most important thing we can give our grandchildren is friluftsliv.” She believes that friluftsliv can be practised and experienced in a good way in the family. General recreational activities for children and young people, for example sports, are well organized and led by other adults. That then makes things quite easy, says Eva. “Did you have a clear strategy that your grandchildren should take part in friluftsliv,” we ask the four pensioners. They all agree and confirm that this is important. Eva responds that “sure, we raise them to enjoy friluftsliv. We know and believe that this is important.” She tells us that the oldest grandchild aged 13 has started going on tenting trips in winter. She feels that this “warms” her heart very much. Similarly, John states that the transfer of friluftsliv to children and grandchildren is a pleasure. He says that he sees this in his oldest son “who is now carrying his children on his back and sets off on walks every weekend. This is the social benefit of it” (raising children to enjoy friluftsliv, our remark). Peter says that experience from and knowledge of friluftsliv “provide the opportunity for the children to take part in what we do because we want them to experience the same that we did.” In this way they have a very deliberate strategy that underlies the idea of taking children on walks to the extent that socialization is an important part of this strategy. Socializing children and grandchildren so they like friluftsliv is important for all our four interviewees.

### “Pure” and valuable friluftsliv experiences based on desire, voluntariness, and motivation

Looking back in time to retrieve values and ideas that may be important for oneself and others in the future corresponds here to harvesting experiences from a lived life. John states that friluftsliv has been very important and has characterized his life in the sense that he and his family have spent much time in nature during holidays and recreation time. John says that “friluftsliv is in this way one of the purest experiences you can have” and that you can get “the world right in your face.” What he is saying is that a person can experience with his senses how weather and wind affect him, and that this is something genuine, and something that represents immutable values which can give insight into both nature and oneself. John elaborates on “pure” experiences, calling them “untainted experiences” and “the way things really are,” that is before the culture and greater society leave their mark on individuals. Does this mean that it is quite easy to find and enjoy these experiences? John states emphatically that the principle for going on trips in nature has always been about “wanting to do it and that it is voluntary.” Going on trips in all seasons because one has a strong desire to do so, without coercion or a sense of obligation, is what has given a lifelong positive relation to the idea of using his body in nature, he claims.

Thus, friluftsliv may seem to be easy to achieve – but which nuances in the experiential value can support the idea of “the good friluftsliv”? In John’s words:

It’s like…you want to have more good experiences…and that’s probably the essence of it…and there is another essence in it too…and that’s if you have never been frozen down to the bone and soaking wet…you’ll never have experienced the great joy of getting dry and warm. And if you have never been hungry and thirsty you have never felt the perfect joy of eating and drinking so you are not thursty …and that means that even the simplest food can be a delight out in nature. Therefore, it’s important to understand the contrasts and to learn to tolerate, because on the other side of wet and cold, hungry and thirsty, wonderful experiences are waiting. So, this is one of the motivations behind wonderful experiences… always…

Friluftsliv thus opens for understanding these contrasts in life. Experiencing contrasts like wet and dry, hungry and full, physical exertion and rest represents feelings that can be gained in friluftsliv. Learning to enjoy friluftsliv is perhaps about feeling these extremes and understanding that experiencing them is beneficial. Mia says that the motivation for friluftsliv can be about mastering. She adds that “the more you master things the more fun it becomes.” Mastering things can also mean having knowledge and competence. Mia believes that friluftsliv competence can be achieved by doing and testing things and in this way gaining experience, an approach she calls testing and gaining experience of “life itself.” John says that wanting to have more good experience is a motivation. Self-confidence is not something you have, John adds, rather something you acquire by subjecting yourself to hardship by “…getting the world in your face.” Gradually these challenges are overcome, you feel well and safe again, and you have mastered something: “That gives you self-confidence.”

### Health promotion and friluftsliv activities

Mia (86) tells us that she walks “…almost 10 kilometres every day” and preferably in nature. While walking you can “see” and experience the world through your body. Mia says that she likes to take walks to experience the light, the seasons, plants and “…everything that is beautiful.” Earlier in life she never considered that she went walking for her health. Now, on the other hand, she thinks more about this, saying that she is “…completely dependent on walking and moving” to stay in shape. She tells us that illness has led to early deaths in her family, and that no other member of her family has lived as long as she has. Thus, she ascribes a crucial role to walking; it has enabled her to live so long as she has. “It’s so obvious that walking is important for your health,” she maintains.

Peter (68) believes that friluftsliv can be a kind of training method. He says that he “keeps things going and is in good physical shape.” On long walks he burns so many calories that he “…loses weight” (without this being a goal in itself). He says that the biological “clock” is ticking faster at his age. He has been diagnosed with an illness, and that “it (life, our remark) can go down the drain.” But he remains active in his friluftsliv to improve his health. He looks after himself and “listens to his body.” He says that he can experience other problems with his body in friluftsliv activities, such as “aches and pains…shoulders and back if you carry a heavy backpack,” but he finds this to be unproblematic. Summing up he says that he has “only positive thoughts about friluftsliv in the context of health.”

John (75) finds that he has reached an age where his movement skills have deteriorated noticeably, but he believes his condition could have been much worse if he did not have “versatile movement experience from before” through friluftsliv. He says that he knows many people at his age “who are unable to walk anywhere” or “do simple things with their bodies.” His versatile movement experiences have been gained through his practising friluftsliv, he says. He adds that the positive health aspect of friluftsliv is to “find joy by being outdoors.” He believes that the combination of using the body and gaining positive experiences is like a “rule without exceptions” in friluftsliv. Both are recognized as ingredients of good health, he says.

Eva (68) offers clear ideas about the link between friluftsliv and health. Physical health is improved by taking walks in nature. Walking in uneven terrain gives good “variation.” She trains strength by “walking with a heavy backpack.” There are social health benefits because “it is easier to talk to people out in nature” than where there is much noise and traffic. She believes that she can talk about other things with the people she walks with in nature than when “sitting and drinking coffee” at home or in a café. She also believes that walking in nature creates a healthy internal “thought process” and therefore benefits mental health. “Thought process” refers to what she is thinking and how she is thinking about things. Her thoughts are different for her when she is walking in nature. Therefore, for her, nature is a “gold mine” for her mental health, she says, adding that she needs to be out in nature to avoid becoming “restless.” She feels she “opens up and does not go into herself” when she is out in nature. Then it is easier to “be released from the internal milling of thoughts” which may be common in everyday life.

Eva also claims that she is “powerfully internally motivated” for being outdoors in nature, and that she gains “self-confidence” from being outdoors. There is so much that captures your attention in nature. Focusing on the joy to be had from movement, such as skating, skiing, walking along a path or along the shore, riding a horse in nature, and being able to see, smell, and think about everything can improve ones’ mental health, she believes. Eva states emphatically that some experiences in nature have given her “happiness.”

This was not always the case for her. Once she suffered from a mental disorder that virtually paralyzed her. She “hit the wall,” was “terrified” about many things, such as losing “functions,” but the worst was that she was unable to get out into nature.

## Discussion

Through the analysis we constructed four themes that describe friluftsliv literacy from childhood to old age and generate understandings of friluftsliv literacy in relation to physical literacy for health throughout the life course. The first theme is that friluftsliv gives experiences in nature in the way that individuals perceive nature qualities and obtain positive emotions in the friluftsliv activity regardless of age or life phase. The stories from the four informants constitute the basis for a phenomenological view of the individual as an active bodily subject in friluftsliv throughout one’s *whole* life. In the previous study, in which we also analyzed the data through phenomenological lenses, we argued that nature interacts with the internal conditions of the students to produce a worthwhile experience, such as self-confidence and self-esteem, in nature [(1), p. 8]. Being an active bodily subject in friluftsliv throughout a whole life, means that the individual is intentional and meaning-forming (39), and that they perceive and experience friluftsliv activity and nature through the body irrespective of age through the various phases of life – from childhood to old age. Special phenomena are sensed and experienced in nature regardless of age and phases of life. Eva, for example, talks about the feeling of true happiness when experiencing the beauty of fluffy light snow crystals. Firsthand experience of and with existential phenomena lead to a sense of identity, and a sense of belonging to nature. Furthermore, within the phenomenological frame we argue that experiences in nature can give much meaning. The four interviewees appreciate being able to enjoy nature. All the pensioners express something about experiences in nature, even if the expressions they use differ. Peter talks about wilderness experiences far from civilization. Mia expresses joy about the “beautiful” landscape. John talks about the genuine authenticity of nature when getting “the world right in your face,” literally feeling the reality of nature whipping your face. Eva believes that she can find “true happiness from the beauty” in nature. The expressions the four have in common are that they appreciate nature and have learnt to interpret incidents and moods from their friluftsliv into their lifeworlds as something valuable through all phases of life.

Another phenomenological dimension is that the subject in nature approaches it, “inhabits” nature [according to Moran (38)], and is in nature because it feels good. Nature will be approached, thus experienced, in an active manner, so that, for example, the trip in the cold winter far north in an area with polar bears can be experienced as very exciting for Peter. John says for example that if you have never been cold and wet, then you have never experienced the great joy of being dry and warm. Friluftsliv provides contrasts, such as wet and dry, hungry and sated, or rest after physical exertion. Experiencing such contrasts and the joy of becoming dry and sated, and resting after exertion, may enable the individual to grow as human beings (3) and to learn to like friluftsliv. Fundamentally the individual has an active approach to nature, and nature is perceived as a meaningful place to be. Being in nature refers to feelings there and then in friluftsliv activities, to differences and contrasts in emotions, and to assigning value to the person you are in nature and in the friluftsliv activity [according to Engelsen (46)]. On the other hand, we are aware that people can see nature as a dangerous place to be and that the individual is more filled with fear than joy and excitement when practicing friluftsliv. We also realize that there are issues of crime and safety for some people to consider going alone or in small groups into nature. However, we argue that experiencing yourself while practising friluftsliv may provide the foundation for self-esteem and an opportunity to enjoy and appreciate the person you are in nature, hence, to like friluftsliv.

A third phenomenological dimension is that the individual’s lived experience in friluftsliv is about an individual’s internal awareness of time and how the past, present, and future are interconnected in an ongoing flow where what is in front of you right now is experienced in relation to a background connected to what has been and what will come in the future for an individual (37). The past, present, and future constitute mutual time horizons in the individual’s consciousness so that, for example, stories and thoughts about friluftsliv in the present can be understood on the basis of previous experiences of friluftsliv. The theory of the past, present, and future constitute mutual time horizons means that memories and previous experiences of friluftsliv can “colour” thoughts in the present in such a way that friluftsliv events and experiences from the past can still have great value. For example, John talks about good nature experiences with others on winter trips where they “spent the night under open skies in the mountains, […] had lit a log fire, and […] looked up at the starry sky.” This can also mean that visions of what will occur in the future in friluftsliv may impact what is appreciated in the present, for example that four people in their day-to-day lives enjoy thinking about friluftsliv with children and grandchildren.

All in all, a phenomenological approach to friluftsliv literacy throughout the life course creates understanding of individual meaning-formation in friluftsliv throughout an entire life. Meaning is formed by being mindfully engaged in doing something of personal importance (39). Meaning-formation is what shifts the individual from one experience to the next, and from an understanding of phenomena experienced in friluftsliv to a new or different understanding [according to Standal (10)]. New perceptions are achieved, and another behavior can be developed. The perception of meaning is also embodied (2), that is to say that in friluftsliv an individual experiences and forms meaning through the body. The body may seek meaning and be the individual’s method of forming meaning in friluftsliv activity and in interaction with other individuals in friluftsliv [according to Standal (10)]. This applies to individuals of all ages, including older adult people.

The second and third themes, which describe friluftsliv literacy through several life phases, are about social values and the transfer of friluftsliv to new generations, as well as “pure” and valuable friluftsliv experiences based on desire, voluntariness, and motivation. To us, it seems to be clear that the interviewees are friluftsliv literate people who are aware of the meaning friluftsliv can have and the socialization that may take place to and in friluftsliv. As mentioned above, Eva says that friluftsliv is “the most important thing” we can give our grandchildren. John says that it is about “pure” experiences that can be understood as important building blocks in the project of raising children and creating a family. The pensioners express, albeit through different words, that socialization is a conscious strategy to influence one’s own children, and in turn grandchildren, and in this way, it makes their life’s work something positive. Friluftsliv for these four pensioners gives meaning – for utility value and enjoyment. The utility value may be the positive contribution to health one gains from friluftsliv and a physically active lifestyle, positive mental thinking about health, and a closely knit family with shared interests and sense of identity. The utility value may also be that an individual, who may be a friluftsliv literate person, has a sustainable awareness (3) and can be an ambassador for being committed to protecting nature [(1), p. 11]. The joy may be found in having a shared project with the family, and perhaps the most important aspect is the adventures to be had in nature, which always occur when people are outdoors, and which are interpreted in very positive terms and with the faith that friluftsliv embodies some of the innermost and most genuine values in life. This joy can also be that they “liked” being outdoors and in nature, in Mia’s words, and that this is the catalyst for learning to like and wanting to transfer important friluftsliv values.

### Contribution to physical literacy for health throughout the life course

The fourth theme is health promotion in friluftsliv activities. The study contributes stories from people who have been active in friluftsliv throughout their long lives in Norway, in the north of Europe, giving the conceptualization of physical literacy for health throughout the life course an interesting nature and friluftsliv dimension. The aspect of health promoting friluftsliv activities corresponds with the view on physical literacy being health promoting (18), suggesting that health-promoting activities may well be practised in nature. Friluftsliv activities may increase physical activity among children, adolescents, adults and the oldest adults, decrease sedentary behavior among both the young and old, and reduce healthcare costs and economic losses associated with physical inactivity in society, which all correspond with elements of physical literacy as health-promoting physical activity (18). This also corresponds with the pragmatic health-determinant and the disease-prevention approaches to physical literacy, which both promise an improvement in health behavior (11). One Norwegian study found that nature experiences and a feeling of home in the wilderness can reduce stress and improve mental health (35). Friluftsliv literacy may improve the health of the population in the way that the Norwegian authorities (presumably authorities in other countries, too) find friluftsliv useful. Even short walks, which may be taken in nature, and a relatively modest number of steps may yield a positive effect, as mentioned above (33).

At the same time, it is important to note that this pragmatic approach to friluftsliv literacy throughout the life course, as well as an idealist-Whiteheadian approach, do not fully consider the new skills that adults need to learn as a result of age-related or health-related conditions connected to functional and mobility changes, as mentioned in the introduction (20). Petrusevski et al. argue that the concept physical literacy should be reconceptualized to include some key components that encompass the characteristics of which aspects of physical literacy are important for adults and the older adult people. These key components are knowledge of age-related changes, and the role of physical activity and being able to self-adapt to physical changes [(20), p. 8156]. This view of Petrusevski et al. can be related to the aspects of friluftsliv literacy in the current study in the way that the concept of friluftsliv literacy also includes some components that encompass the characteristics important for physical literacy for adults and the oldest adults, such as reduced mobility and physical constraints. At the same time, friluftsliv literacy is about the freedom to choose the activity to be practised, as well its scope, place and time in accordance with the individual’s background, interests, wishes, and motivation. Friluftsliv activities can be adapted to individual backgrounds, and different ages and phases of life, as long as the individuals can decide themselves. The stories told by the four pensioners show how friluftsliv is adapted to age and phases of life and that the individual can self-adapt to physical changes and still move with poise, confidence, and motivation (2) in friluftsliv in one’s senior years, with simple adaptations. Here, freedom, flexibility, and autonomy are key concepts. Important dimensions of friluftsliv literacy throughout the life course are therefore the freedom to choose, flexibility of friluftsliv activities, and the individual’s autonomy enabling one to adapt friluftsliv activities to individual capacities.

## Conclusion

With a constructionist epistemology as the frame that led us into our study, we put ourselves in motion as researchers and interviewed older adult people about friluftsliv as a phenomenon in their lifeworlds and about friluftsliv literacy as a concept. We developed four themes from the data material that describe friluftsliv literacy throughout the life course and that generate understandings of friluftsliv literacy in relation to physical literacy for health throughout the life course. The themes of friluftsliv literacy from childhood to old age should be understood with some reservation relating to themes generated in this way, but they may be valid beyond themselves within the epistemological frame we chose for the study. They may also be building blocks for further knowledge development. These four themes elaborate concepts from previous research; experience in nature, the subject in nature, identity formation, self-confidence, and sense of social fellowship, and help us to understand friluftsliv literacy from a wider perspective as they are related to friluftsliv literacy throughout the life course. We argue that friluftsliv literacy for adults and the oldest adults promotes nature experiences through the *whole life*, and the concept offers a view on the active subject in friluftsliv through the various life phases. Moreover, friluftsliv literacy promotes “pure” friluftsliv experiences based on internal motivation and desire, voluntariness, and freedom. In addition, we argue that throughout the life course friluftsliv literacy promotes important social values, especially within family contexts, social values that enhance the quality of life. We experienced through the interviews with Mia, John, Peter, and Eva that it is highly meaningful to pass friluftsliv values on to new generations in the family so that children and grandchildren also learn to like friluftsliv.

The concept friluftsliv literacy throughout the life course contributes to the conceptualization of physical literacy for health in the way that it promotes people learning to like friluftsliv at a young age, an attitude that can then be nurtured and maintained throughout one’s entire life by practising friluftsliv and adapting to individual capacities and life phases. Friluftsliv literacy promotes the individual’s physical, mental, and social health, and contributes to improving the health of the general population. However, the stories from the four interviewees reflect that understandings of friluftsliv literacy in relation to physical literacy for health throughout the life course comprise an approach to nature that is more existential. Friluftsliv informs the individual’s identity and is the basis for seeing oneself as a friluftsliv literate person. Friluftsliv literacy from childhood to old age thus includes an existential phenomenological and idealist dimension in the form of self-esteem, a sense of companionship with nature, and identity, including family identities. For many Norwegians, going for a walk in nature, in general, or trekking to a mountain top, in particular, are expressions of such values as courage, strength, and stamina, and as something that promotes health, where one might also believe that friluftsliv promotes learning skills and physical activities that have positive effects on health, such as learning to ski, hiking in the woods and mountains, or paddling a canoe on a lake. But in reality, there is much more of something else. This “other” aspect includes learning to like friluftsliv as your parents and grandparents have done, where friluftsliv gives meaning in a life-long perspective – for utility and pleasure, whatever one’s age.

## Data availability statement

The raw data supporting the conclusions of this article will be made available by the authors, without undue reservation.

## Ethics statement

Written informed consent was obtained from the individual(s) for the publication of any potentially identifiable images or data included in this article.

## Author contributions

ES: Conceptualization, Data curation, Formal analysis, Investigation, Methodology, Writing – original draft. IL: Conceptualization, Formal analysis, Methodology, Writing – original draft.
